# Simulation Tool and Online Demonstrator for CDMA-Based Ultrasonic Indoor Localization Systems

**DOI:** 10.3390/s22031038

**Published:** 2022-01-28

**Authors:** María Carmen Pérez-Rubio, Álvaro Hernández, David Gualda-Gómez, Santiago Murano, Jorge Vicente-Ranera, Francisco Ciudad-Fernández, José Manuel Villadangos, Rubén Nieto

**Affiliations:** 1 Department of Electronics, University of Alcalá, 28801 Alcalá de Henares, Madrid, Spain; alvaro.hernandez@uah.es (Á.H.); santiago.murano@edu.uah.es (S.M.); jorge.vicenter@uah.es (J.V.-R.); francisco.ciudad@uah.es (F.C.-F.); jm.villadangos@uah.es (J.M.V.); 2 Signal Theory and Communications Department, Rey Juan Carlos University, 28942 Fuenlabrada, Madrid, Spain; david.gualda@urjc.es; 3 Electronics Department, University of Patagonia San Juan Bosco, Comodoro Rivadavia (Chubut), Tierra del Fuego V9410 AQD, Argentina; 4 Electronics Technology Department, Rey Juan Carlos University, 28933 Móstoles, Madrid, Spain; ruben.nieto@urjc.es

**Keywords:** simulation platform, Ultrasonic Indoor Positioning System (UIPS), online demonstrator, encoding techniques for active sensing

## Abstract

This work presents the CODEUS platform, which includes a simulation tool together with an online experimental demonstrator to offer analysis and testing flexibility for researchers and developers in Ultrasonic Indoor Positioning Systems (UIPSs). The simulation platform allows most common encoding techniques and sequences to be tested in a configurable UIPS. It models the signal modulation and processing, the ultrasonic transducers’ response, the beacon distribution, the channel propagation effects, the synchronism, and the application of different positioning algorithms. CODEUS provides results and performance analysis for different metrics and at different stages of the signal processing. The UIPS simulation tool is specified by means of the MATLAB© App-Designer environment, which enables the definition of a user-friendly interface. It has also been linked to an online demonstrator that can be managed remotely by means of a website, thus avoiding any hardware requirement or equipment on behalf of researchers. This demonstrator allows the selected transmission schemes, modulation or encoding techniques to be validated in a real UIPS, therefore enabling a fast and easy way of carrying out experimental tests in a laboratory environment, while avoiding the time-consuming tasks related to electronic design and prototyping in the UIPS field. Both simulator and online demonstrator are freely available for researchers and students through the corresponding website.

## 1. Introduction

The global spread and huge increase in new Location-Based Services (LBS) or context-aware computing, in which accurate information about the target location is required, have boosted the research in Indoor Positioning Systems (IPSs) in different application domains: museums and tourism [1,2], gaming and augmented reality [3], mall navigation [4], hospitals and monitoring of the elderly [5], airports [6], logistics or security, and emergency responders [7]. In general, IPSs can be divided into two main categories: infrastructure-free and infrastructure-based. The first includes Inertial Measurement Units (IMUs) [8], and vision-based [9] or magnetic-based systems [10]. These are often flexible enough to properly work in any environment, without requiring any previous adaptation. In other cases, it is necessary to have some previous knowledge about the environment. By comparison, infrastructure-dependent systems commonly take advantage of signals of opportunity or may require the installation of dedicated infrastructure. Among this range, Radio Frequency (RF)-based systems are widely used because they can apply the WiFi infrastructure already installed for communication purposes to positioning after a calibration process (fingerprinting) [11], although with a typical meter-level accuracy [12]. Other alternatives are either Bluetooth Low Energy (BLE), based on inexpensive and long-battery-life antennas for a zone-based approach [13], or Ultra-Wideband (UWB) technologies to obtain accuracies below decimeters [14,15]. In this context, some relevant surveys about IPS and their associated technologies can be found in [16,17,18]. Note also that different technologies may be complementary for indoor positioning, obtaining hybrid systems that increase coverage, reliability, and confidence. Usually, these systems use a technology that provides absolute ranging (e.g., WiFi, RF, and cameras) and another that provides relative ranging (inertial sensors) [19,20,21], using probabilistic filters (e.g., a Kalman filter [22]) for merging the data.

Acoustic-based systems such as Ultrasonic IPSs (UIPSs), which are the focus of this work, are a cost-effective solution that can reach accuracies in the range of centimeters with coverage distances up to some tens of meters. This accuracy is usually obtained using beacons with encoded transmissions and pulse compression techniques for the detection at the receiver, which also ensures a high robustness to noise and more tolerance to other negative effects [23,24]. Furthermore, ultrasonic transducers are small, their transmissions are room-constrained, and they can be easily integrated into mobile phones [25].

The final performance of an UIPS is determined by some factors; in fact, there is a wide variety of available options regarding equipment, beacon deployment, signal configuration, or positioning methods. In this regard, there is a trade-off between the minimum number of beacons to be installed to reduce costs, their geometric configuration to obtain a suitable coverage area, and the redundancy required to cope with possible occlusions. One alternative is to carry out a deployment with ultrasonic beacons only in zones where centimeter positioning is required, while using other technologies, such as inertial sensors or RF signals available in the environment, in those areas where a coarse-grained positioning is enough [25]. Beacons can emit simultaneously or at certain intervals using a time division multiple access (TDMA) technique. TDMA has the advantage of reducing the Multiple Access Interference (MAI), but decreases the position update rate, which can limit the detection of a target in motion. A common strategy to allow simultaneous emission while minimizing the MAI is the incorporation of broadband sequences from Code Division Multiple Access (CDMA) techniques. This implies assigning a different and unique waveform to each emitter, in such a manner that it can be effectively separated from the other transmissions at the receiver, even in the presence of noise [24,26]. It is worth mentioning that in ultrasonic positioning, bursting emissions are more common because they deal better with multipath interferences, at the expense of worsening the correlation properties of many sequences that are intended for periodic correlation. The bandwidth restrictions of the ultrasound transducers and the modulation scheme for the signal transmission strongly affect the aforementioned correlation properties, thus having an impact on the final system performance [23]. In addition, on the receiver side, decisions must be made about the microphone to be used, the sampling frequency, the synchronism constraints, or the implemented positioning algorithm. Spherical trilateration can be used in UIPS, based on Time of Arrival (ToA) measurements; nevertheless, in this case, it is necessary to provide some synchronization between the beacons and the target receiver. To avoid adding any technology apart from the ultrasonic signals, Time-Differences-of-Arrival (TDoA), which requires the inclusion of another beacon, can be used for 3D positioning. This hyperbolic trilateration often results in lower accuracies compared to the spherical option [27], but does not require a synchronism link between the beacons and the receiver.

It is well known that experimentally analyzing the influence of the aforementioned parameters in a real UIPS may be time consuming and arduous [28]. Thus, software simulation models are useful to obtain a full picture of how the system will behave under different circumstances. Furthermore, a predefined experimental UIPS architecture, which is flexible enough to allow the testing of different encoding techniques, modulations, or transmission schemes, can become key to obtain preliminary results with real signals, while avoiding the big efforts and hardware/software skills demanded by the implementation and prototyping of any electronic system. If this predefined architecture can be remotely configured and is accessible to the research community, it will allow the evaluation and comparison of different configurations and algorithms under the same conditions. Such an approach is desirable because the variety of beacon deployments and equipment used in experiments is so wide and customized that undertaking a fair comparison of the algorithms proposed by different research teams is not feasible [18,29]. 

This work presents the CODEUS platform, which is oriented to facilitate, by both simulation and preliminary experimental tests, the comparison of different encoding and modulation schemes in a typical UIPS scenario, by means of a user-friendly interface. The tool allows the convenient avoidance of the initial gap of researchers in experimental tests, and is an educational tool for PhD students working in the UIPS field. The main contributions of this work are:

- Integration in a single simulation platform of a large variety of real-world issues and configurable parameters that affect the design and behavior of acoustic positioning systems. The platform also deals with the high-level processing stage regarding peak detection and positioning estimation.
- Design of a remotely configurable UIPS that can be managed from a website to obtain preliminary experimental results, and to compare diverse signal designs by different authors under the same setup.
- Validation of simulated results against the corresponding experimental tests obtained with the online demonstrator.

The remainder of this manuscript is organized as follows: Section 2 reviews the related work; Section 3 presents the CODEUS platform overview; Section 4 is dedicated to the simulation tool, whereas Section 5 deals with the description of the online demonstrator; Section 6 describes a practical case with the online demonstrator and compares the results with those obtained from the simulator; and, finally, conclusions are discussed in Section 7.

## 2. Related Work

Simulations have become a key tool to deal with the design, testing, and validation of positioning systems, and several approaches can be found in the literature [30]. SMILe [31] is an open-source tool for developing and evaluating indoor RF positioning methods based on ToA or TDoA. The simulator is developed under OMNET++, which is an extensible component-based C++ framework and simulation library for building network simulators, and also includes the INET open-source model library from the OMNET++ environment to build complex wired and wireless communications. It allows the user to adjust different aspects of the simulation, such as radio wave propagation models, node mobility, or hardware clock accuracy. In addition, an analytical package was written in Python 3 to assist the user in the implementation and evaluation of ToA and TDoA solvers. PyLayers [32] is a Python platform that provides a flexible tool to investigate radio propagation channel and indoor localization algorithms using UWB radio signals. It is based on a ray-tracing model, and also comprises an indoor human mobility simulator for wearables and WBAN. The Navindoor platform is more versatile, although with less complex ToA models [33]. It is also an open-source tool, developed under object-oriented programming in MATLAB©, which allows the definition of the scenario, trajectory, and dynamics of moving people in indoor spaces, including the synthetic generation of different types of signals: beacon-based (Received Signal Strength, ToA, and Angle-of-Arrival) or beacon-free (barometer, magnetometer, inertial, and gyroscope), and their associated processing for the comparison of the resulting estimations. In Young et al. [34], IMUsim is presented as a simulation environment developed to model the aspects of IMU operation. It is based on Python under an open-source license, ready to be used with new models and algorithms, thus allowing continuous development. This simulator has been focused on inertial applications and can simulate the corresponding IMU measurements from the trajectories defined by any application.

The work in Álvarez et al. [35] specifically addresses the modeling of a beacon-based UIPS in MATLAB, considering many aspects and details in the low-level processing of ultrasonic signals, such as the effect of the beacons’ frequency response, the bandwidth, the multipath propagation, or the receiver’s movement. The system is based on Binary Phase Shift Keying (BPSK)-modulated Kasami codes and provides results in terms of ToA measurements and quality of the correlation functions (sidelobe-to-mainlobe ratio and shift of the auto-correlation peaks). Other works have attempted to optimize the ToA estimations through simulation and experimental tests of different encoding and modulation techniques. Examples of BPSK modulations with different digital codes can be widely found in the literature [23,36,37]. Most of these codes are designed for periodic correlation, and they present higher correlation sidelobes in case of aperiodic transmissions. By comparison, codes that operate on a multiple-sequence-per user basis, such as Complementary Set of Sequences (CSS) [38], provide ideal properties in the sum of the aperiodic correlation functions. Nevertheless, they may also require more complex modulation or transmission schemes, which often imply a degradation in their initial correlation properties [39]. Generalized Orthogonal codes, such as Loosely Synchronous (LS) codes, are unitary sequences that have also been used in UIPS, due to the Zero Correlation Zone (ZCZ) that appears around the main correlation peak; however, they are constrained to quasi-synchronous applications where the different incoming signals must be received within this ZCZ [40,41]. Furthermore, LS codes, in addition to CSS, do not cope well with the Doppler effect [42]. Chirp signals have often been involved due to the suitable properties of their compressed pulse against Doppler, but their performance is constrained in simultaneous multiuser environments [43]. Polyphase sequences, such as Zadoff-Chu codes [44], can be a suitable option due to their length flexibility and tolerance to Doppler. In Murano et al. [45], different Quadrature PSK (QPSK), Orthogonal Frequency Division Multiplexing (OFDM), and Orthogonal Chirp Multiplexing (OCDM) modulation schemes are compared for the transmission of Zadoff-Chu codes in an UIPS. Moreover, the Frequency-Hopping Spread Spectrum (FHSS) has been applied to IPSs to handle multiple access [46]; however, ultrasonic transducers have a narrow frequency bandwidth, so the effective bandwidth for every hop is significantly reduced when the number of simultaneous emissions increases. Taking into account the previous considerations, it can be stated that there is no unique solution for the wave design of a UIPS, and obtaining the highest possible robustness and accuracy depends on the UIPS configuration and application [41,47].

Regarding the aforementioned context, this work presents a simulator that covers all the processing levels of the ultrasonic signal, in addition to an online demonstrator, thus helping reduce the barriers to real-world validation. As an example of usability, the proposed platform has been involved in the Local Positioning Systems course from the Master in Electronic Engineering at the University of Alcalá, in addition to the Indoor Positioning and Indoor Navigation Conference (IPIN 2021) as part of Tutorial 4: Tools for Acoustic Indoor Localization Systems. The simulator helped bridge the gap between the equations and algorithmic definitions and the practical implementations required in a successful learning environment. Thus, the theoretical coaching was enhanced with a practical approach, in which attendants could easily simulate their own UIPS and test their positioning-related algorithms with real signals. The discussions and suggestions after the practical tutorial in IPIN 2021 contributed to improving the platform. As a result, a new peak detection algorithm was included to provide multipath compensation and more flexibility was enabled. As a possible indicator of the interest of the research community in Acoustic IPS (AIPS), Table 1 shows the percentage of papers related to AIPS in the last five editions of the IPIN conference. It can be observed that acoustic technology is still important in the field of IPS.

## 3. CODEUS Platform Overview

**Figure 1:**
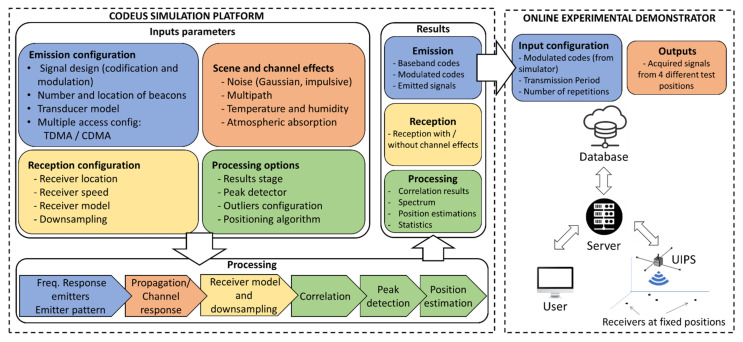
General block diagram of the CODEUS platform: simulator and online demonstrator.

CODEUS includes a simulator and an online demonstrator for a UIPS based on encoding techniques. The system is based on a set of active encoded beacons located at fixed positions in the environment, and a receiver that processes the incoming signals determines the ToA (or TDoA if it is not synchronized with the beacons), and estimates its own position. Figure 1 illustrates the global block diagram of the proposed platform. The simulation model is highly configurable, because the user can define the number and location of beacons, the codes/modulation for the signal design, the type of transducer, or the medium access technique for beacons (i.e., time-multiplexed, among others). On the receiver side, it allows configuration of the location and type of receiver, its moving speed, the sampling frequency in the acquisition, and the definition of the synchronism with the emitting beacons. Furthermore, it includes other typical effects of the ultrasonic signal propagation, such as multipath, noise, temperature, or air absorption. Regarding the position computation, several algorithms can be tested, depending on the selected spherical or hyperbolic trilateration. Both numerical and graphical results are shown and can be downloaded at every stage of the processing. The modulated codes are easily exported by means of the simulator user interface and they are already adapted for experimental tests in the proposed online demonstrator, thus obtaining results with real signals that can be compared with those from the simulator. The demonstrator is based on a UIPS formed by five emitters placed at a height of 348 cm in a 70.7 cm × 70.7 cm square. It provides a coverage area of approximately 30 m^2^ on the floor, where four receivers are placed at certain locations of interest in a laboratory environment. Any user can remotely access and configure the UIPS from a website, and the experimental acquisitions will be available to be downloaded from the cloud. Both the online demonstrator and simulator are freely available for researchers through the website available online: http://www3.uah.es/locate-us/ (accessed on 23 December 2021), after a basic registration process. 

## 4. Simulation Platform

A detailed description of the proposed simulation platform is provided hereinafter, by dealing with the different modules that should be set up for a correct operation: the emission module (beacons), the receiver, the channel modeling, the positioning algorithm used to estimate the receiver’s position, and the display of the final results. The MATLAB© App-Designer environment was used to incorporate the user interface and integrate the developed algorithms. The MATLAB© environment is widely used both by researchers and educators due to its signal processing potential, flexible computational possibilities, and options to create new tools, where new functionalities or updates can be easily included [33,48,49].

### 4.1. Emission Configuration

**Figure 2:**
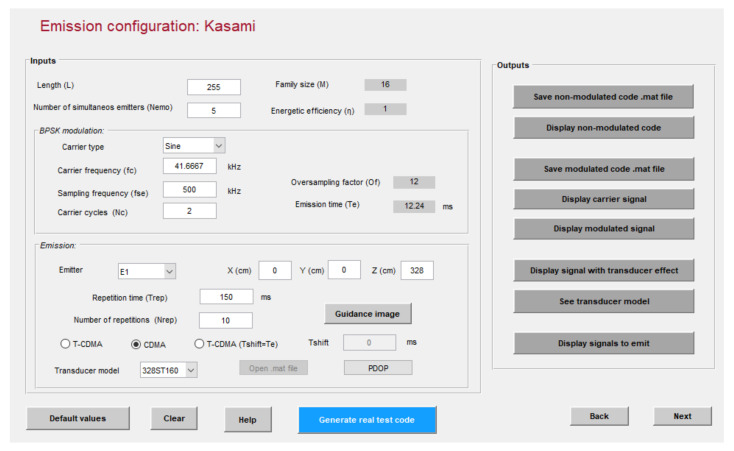
Example of emission configuration with five 255-bit Kasami codes modulated in BPSK with two carrier cycles at 41.67 kHz and a sampling frequency of 500 kHz.

The first step in the emission configuration is the code selection, and the second step allows the design of the different parameters related to the emission. An example for the configuration of Kasami codes can be observed in Figure 2. Note that Kasami sequences have already been considered as a suitable option to encode ultrasonic transmissions, so they will be used in most examples hereinafter.

**Figure 3:**
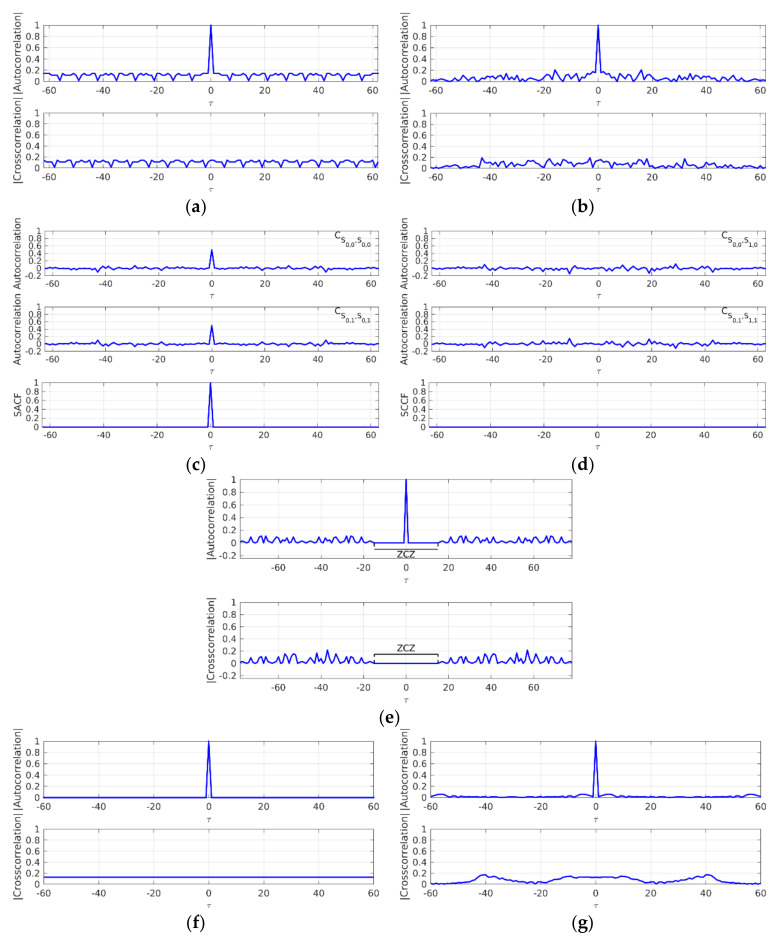
Correlation functions for those sequences considered in the CODEUS platform: (**a**) normalized periodic and (**b**) aperiodic correlation functions of Kasami sequences with length *L_Kas_* = 63; (**c**) SACF and (**d**) SCCF for CSS with *M_CSS_* = 2 and *L_CSS_* = 64; (**e**) normalized aperiodic correlation function of LS sequences with length *L_LS_* = 79 and Zero Correlation Zone around the origin *ZCZ* = 15; (**f**) normalized periodic and (**g**) aperiodic correlation function for Zadoff-Chu sequences with *L_ZC_* = 61.

As mentioned above, the optimization of the signals transmitted by the beacons allows the accuracy and robustness of the ranging estimation to be enhanced. The correlation of the received signal with the emitted pattern should be as close as possible to a Kronecker delta and, in multiuser applications, the cross-correlation functions among the different emission sources should be zero. In recent decades, many encoding schemes have been proposed in UIPSs, each with their corresponding correlation properties [26] and generation methods, which can be computationally expensive. The proposed platform automatizes the generation of sequences and incorporates the most common of these in the ultrasonic signal design: Kasami sequences [50], Complementary Set of Sequences (CSS) [38,51], Loosely Synchronous codes (LS) [40], and Zadoff-Chu codes (ZC) [44,52]. Figure 3 shows the difference between aperiodic and periodic correlations for the proposed schemes. For more flexibility, the CODEUS platform allows the testing of any other encoding scheme in addition to the previous ones, by uploading a configuration file with the corresponding transmissions to be carried out.

**Figure 4:**
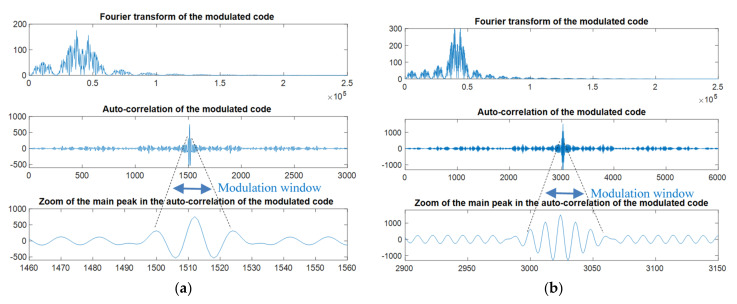
Effect of the BPSK modulation in the auto-correlation function of a 63-bit long Kasami code with *f*_c_ = 41.67 kHz, *f*_s_ = 12 and: (**a**) *O*_c_ = 2; (**b**) *O*_c_ = 4.

Modulation is required to adjust the codes to be transmitted to the bandwidth available in the transducers. The modulation technique affects the correlation results from the matched filtering detection; hence, some authors have analyzed the suitability of different schemes [43,44,45,46]. Phase Shift Keying (PSK) modulations, such as Binary Phase Shift Keying (BPSK) or Quadrature Phase Shift Keying (QPSK), are widely used in UIPSs and are the option included by default in the simulation platform (BPSK for binary sequences and QPSK for complex sequences). Other options can also be tested by customizing the sequence design. The main advantages of PSK modulations are their simplicity and efficient use of the transducer bandwidth; nevertheless, they also cause some sidelobes in the surroundings of the main correlation peak, as can be observed in Figure 4, which may imply an error in the ToA determination. The PSK modulation of a sequence *s_k_*[*l*] is described in Equation (1):

(1) $$\begin{array}{c} Cr\left[n\right]\;=\;\cos\left(2\pi f_{c}n\right)\;-\;j\;\cdot \;\sin\left(2\pi f_{c}n\right);\;0\leq \frac{n}{f_{s}}\leq \frac{O_{f}O_{c}}{f_{s}}\\ m_{k}\left[n\right]\;=\;\sum_{l=0}^{L_{c_{k}}-1}\left[\mathbb{R}\left(s_{k}\left[l\right]\right)\;\cdot \;\mathbb{R}\left(Cr\left[n\right]\right)\;-\;Im\left(s_{k}\left[l\right]\right)\;\cdot \;Im\left(Cr\right)\right] \end{array}$$

where *Cr*[*n*] is the complex carrier for the QPSK modulation (note that if the sequence is binary, the imaginary part will be zero and the carrier will only have the real part, resulting in a BPSK scheme); *O_c_* is the number of carrier cycles; *O_f_* = *f_c_*/*f_s_* is the oversampling factor, or ratio between the carrier frequency *f_c_* and the sampling frequency *f_s_*; and *m_k_* is the modulated signal to be emitted.

**Figure 5:**
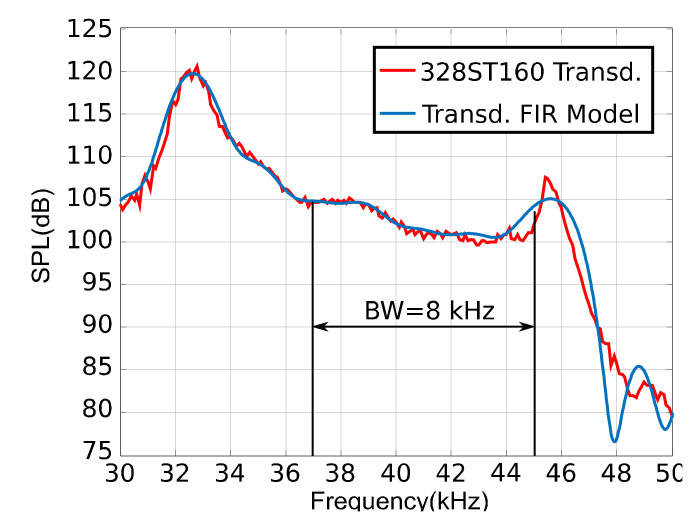
328ST160 transducer frequency response, measured (red) and the FIR filter model (blue) [45].

The platform allows the transducer models used in the emission or reception to be inserted. It also provides some preloaded models that can be selected from a list box: the Prowave 38ST160 [53] in the emission stage; and, in the reception stage, the GRAS40BE [54] or a MEMS microphone SPU414HR5HSB [55]. A FIR filter was designed to model the transducers’ effect; in this way, Figure 5 shows the measured transducer frequency response for the Prowave 38ST160 (in red), compared with the modeled one (blue line).

**Figure 6:**
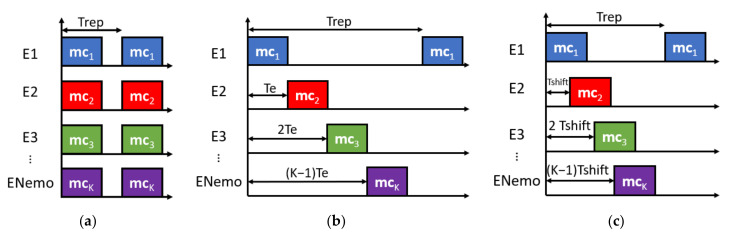
Configuration of the multiple emission scheme: (**a**) CDMA, (**b**) TCDMA (*T_shift_* = *T_e_*), and (**c**) TCDMA with configurable *T_shift_*.

The simulation platform allows different techniques for multiple access, as can be observed in Figure 6. In all of these, every emitter is assigned a unique modulated code *m_k_*, so the interference among them is minimized using codes with low cross-correlation functions. In the CDMA case, all the transducers emit simultaneously. A Time Code Division Multiple Access (TCDMA) case that combines the advantages of both TDMA and CDMA is also possible, where the transducers emit consecutively over time, one after other, and each one transmits its corresponding code. Finally, the transducers can emit with a configurable separation *Tshift* from the others, so any overlap can be simulated (regardless of whether *Tshift* equals the time *T_e_* required for the emission of every code, the TCDMA method is obtained). The platform allows the configuration of the emission repetition period, in addition to the number of emissions to be performed, which can be useful for obtaining statistical comparisons.

Another key aspect is the determination of the number and location of beacons that compose an UIPS, since this strongly affects the cost, coverage area, and accuracy of the final system. The Position Dilution of Precision (PDOP) [56] is a common metric used to select the number and distribution of the beacons, as it shows the uncertainty of the receiver’s location estimation at different positions according to the noise in the measurements and the location of the beacons. As can be observed in Figure 2, the position of beacons can be defined in 3D coordinates (*x*, *y*, and *z*) in the simulator environment, and the PDOP can also be displayed. The parameters that can be configured to analyze this metric are: the height of the receiver; the size of the positioning volume; the interval between the points that form the grid; and the standard deviation of the Gaussian noise related to the distance measurements, considered as a constant value for all the points in the positioning volume, because the coverage of an ultrasonic location system is reduced.

For each evaluated position in the grid, the PDOP value is obtained according to Equation (2):

(2) $$PDOP=\frac{\sqrt{\sigma_{x}^{2}+\sigma_{y}^{2}+\sigma_{z}^{2}}}{\sigma_{m}}$$

where $\sigma_{x}^{2}$, $\sigma_{y}^{2}$ and $\sigma_{z}^{2}$ are the values of the covariance matrix C, which is obtained according to Equation (3); and $\sigma_{m}$ is the standard deviation of the Gaussian noise related to measurements.

(3) $$C=\sigma_{m}^{2}\;\cdot \;{\left(J^{T}\cdot J\right)}^{-1}$$

The Jacobian matrix J is obtained according to Equation (4):

(4) $$J=\left(\begin{array}{ccc} \frac{\partial f_{1}}{\partial x} & \frac{\partial f_{1}}{\partial y} & \frac{\partial f_{1}}{\partial z}\\ \frac{\partial f_{2}}{\partial x} & \frac{\partial f_{2}}{\partial y} & \frac{\partial f_{2}}{\partial z}\\ \cdots & \cdots & \cdots \\ \frac{\partial f_{n}}{\partial x} & \frac{\partial f_{n}}{\partial y} & \frac{\partial f_{n}}{\partial z} \end{array}\right)$$

where $f_{n}$ is the $n^{th}$ measurement from the nth beacon, represented either by the Euclidean distance between the position of the receiver and the nth beacon in the spherical case (assuming that emitters and receivers are synchronized) shown in Equation (5), or by the difference in Euclidean distances shown in Equation (6), using one emitter as a reference, in the case in which there is no synchronization between emitters and the receiver (hyperbolic solution).

(5) $$f_{n}=\sqrt{{\left(x-x_{n}\right)}^{2}+{\left(y-y_{n}\right)}^{2}+{\left(z-z_{n}\right)}^{2}}$$

(6) $$f_{n}=\sqrt{{\left(x-x_{n}\right)}^{2}+{\left(y-y_{n}\right)}^{2}+{\left(z-z_{n}\right)}^{2}}-\sqrt{{\left(x-x_{n}\right)}^{2}+{\left(y-y_{n}\right)}^{2}+{\left(z-z_{n}\right)}^{2}}$$

where $\frac{\partial f_{n}}{\partial x}$, $\frac{\partial f_{n}}{\partial y}$ and $\frac{\partial f_{n}}{\partial z}$ are de derivatives of the nth measurement with respect to the coordinates of the receiver.

**Figure 7:**
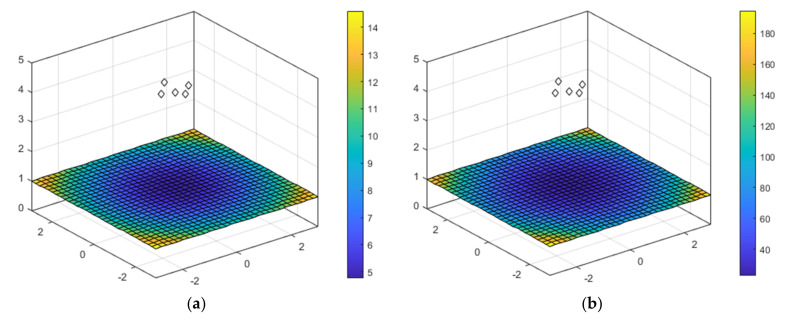
Study of the PDOP considering a 6 m × 6 m area in the XY plane, and five beacons placed at a height of 348 cm, in a 70.7 cm × 70.7 cm square structure: (**a**) spherical; (**b**) hyperbolic.

Figure 7 shows a PDOP simulation for the spherical case (a) and the hyperbolic case (b), setting the area under analysis in the XY plane with a 6 m long side, with a resolution grid of 0.10 m, and a receiver’s height of 1 m. Note that the example uses the same UIPS configuration as the online demonstrator, which is formed by five beacons placed at a height of 348 cm in a 70.7 cm × 70.7 cm square, and which have been represented with a white rhombus in Figure 7. This distribution is also the one used by default in the simulator.

As can be observed in Figure 7, the lowest values of PDOP in both cases are in the center of the area, so in these locations the uncertainty related to the estimation of the receiver’s position is reduced in comparison with corners, where the PDOP values are high. Another consideration to take into account is that the uncertainty is higher in the hyperbolic case than in the spherical case; nevertheless, the hyperbolic case has the advantage of not requiring synchronization between the emitters and the receiver.

### 4.2. Receiver Configuration

**Figure 8:**
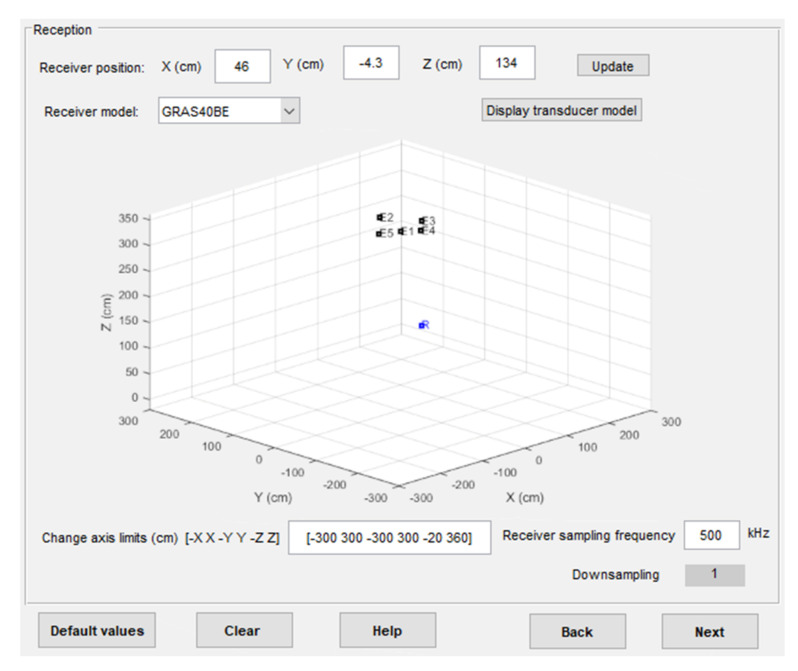
Example of a scene configuration in the simulator environment.

Figure 8 shows a screenshot of the software for the scene configuration, where the position and model of the receiver can be selected, in addition to the sampling frequency at the reception. Thus, a downsampling factor can be simulated to consider the case in which the acquisition system has memory constraints.

### 4.3. Channel Modeling

**Figure 9:**
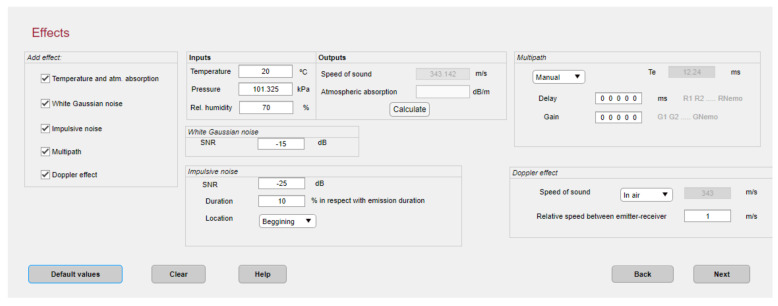
Configuration of different channel phenomena in the CODEUS simulator environment.

The channel model considered hereinafter is based on applying the widely used ray tracing [57,58] to randomly model the multipath effect coming from an indoor environment, resulting in detecting multiple copies of the beacons’ transmission at the receiver. Together with this ray tracing, the CODEUS platform also allows a set of typical effects in air positioning systems to be inserted, such as Gaussian and impulsive noise, temperature, atmospheric absorption, and Doppler effect. All these parameters can be observed in Figure 9. The beacons transmit their corresponding modulated signal *m_k_*(*t*) that will be affected by the transducer response $T_{h}$ and the noise $\eta \left(t\right)$. At the reception, all the signals coming from the beacons are received, each with its corresponding propagation delay $t_{p_{k}}$, transmission delay $t_{t_{k}}$ due to the multiple access technique, and attenuation $A_{k}$, as indicated in Equation (7).

(7) $$r\left(t\right)\;=\;\overset{K_{}}{\underset{k=1}{\sum }}A_{k}\cdot \left[T_{h}\left(t\right)\;*\;m_{k}\left(t-t_{p_{k}}-t_{t_{k}}\right)\right]+\eta \left(t\right)$$

where the operator ∗ denotes the convolution and the index 1 ≤ *k* ≤ *K* indicates the number of the emitter. Note that, in a CDMA scheme, all the emitters transmit simultaneously, so $t_{t_{k}}=0$; by comparison, in TCDMA schemes, an emitter transmits during a specific slot time that is modeled by this parameter $t_{t_{k}}$.

The White Gaussian Noise (AWGN) added to the emitted signal considers the energy measured from the modulated signal to insert white noise and accomplish the desired Signal to Noise Ratio (SNR) value. If the impulsive noise is also selected, then three parameters must be fulfilled: the SNR (normally smaller than the Gaussian noise), the duration of the noise specified as a percentage of the signal duration, and its location over time.

Another interesting effect involved here is the multipath, related to the multiple delayed and attenuated replicas of the transmitted signal that reach the receiver due to the specular reflections from walls and objects. The simulation platform allows three configuration options: Manual, where one delay (in ms) and gain per emitted signal is considered; a Random option that inserts two randomly delayed multipaths, with a gain exponentially attenuated according to the value of the delay; and a more Realistic option that considers the impulse response of the room through the image method described in [35,57].

The estimated ToAs or TDoAs depend on the distance between the emitter and the receiver, and on the propagation speed of sound in air, which is influenced by the temperature [59] as follows:

(8) $$c=c_{0}\cdot \sqrt{1+\frac{T\left[{}^{\circ}C\right]}{273.15}}$$

where $c_{0}=331.6\;m/s$ is the speed at 0 °C and T is the air temperature. The CODEUS platform also considers the atmospheric absorption in dB/m, which depends on the temperature, atmospheric pressure, and humidity. 

Finally, the Doppler shift related to the relative movement between the receiver and the beacons is also considered. This effect is modeled by assuming a virtual sampling frequency $f_{s}^{´}$ for the emitted signal as follows [45]:

(9) $$f_{s}^{´}=f_{s}\left.\left[1-\frac{\vec{v_{r}}}{c}\right.\right]$$

where $\vec{v_{r}}$ is the receiver velocity vector. The signal acquired by the receiver at the actual sampling frequency $f_{s}$ is obtained by an interpolation and decimation process.

### 4.4. Positioning Algorithm

The proposed platform allows the position of the receiver to be estimated using two possible methods: Gauss–Newton (GN) [60] or Cayley–Menger (CM) [61] in the spherical case; and GN in the hyperbolic case. The GN method is based on the use of the Jacobian matrix shown in Equation (4) to solve the position of the receiver, by finding a minimum value of the vector ∆X, as a result of an iterative process.

(10) $$∆X={\left(J^{T}\;\cdot \;J\right)}^{-1}\;\cdot \;J^{T}\;\cdot \;B$$

where B is a vector of the differences between the measurements (distances or difference of distances) and the estimation of those measurements for each algorithm iteration (Equation (11)).

(11) $$B=\left(\begin{array}{c} {\hat{f}}_{1}-f_{1}\\ {\hat{f}}_{2}-f_{2}\\ \cdots \\ {\hat{f}}_{n}-f_{n} \end{array}\right)$$

In the case of the CM method, the estimation of the position is based on the use of Cayley–Menger determinants [62], so the solution is geometric. Here, the number of emitters used must be constant and set at four for 3D position estimation. This method is more complex than the previous one; further details can be found in [61].

**Figure 10:**
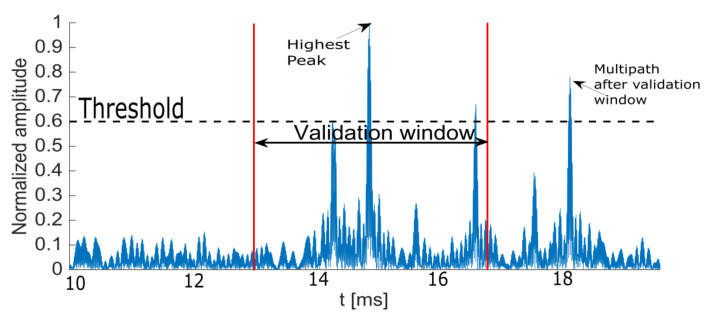
Example of a non-valid measurement after the peak detection algorithm.

It is important to note that the positioning algorithm uses the measured distances after a filter at the correlation phase (before obtaining the distances from the ToAs or TDoAs measurements). This filter discards measurements strongly affected by the multipath: it identifies the highest correlation peak and sets a configurable validation window around this main peak. A threshold defined by the user is also considered to identify more peaks. If any peak appears outside of the validation window and exceeds the threshold, the measurement from that beacon is discarded; see Figure 10 as an example of the mentioned filter. More flexibility regarding the peak detection algorithm will be included in the future to deal with multipath situations [63]. 

### 4.5. Results and Merit Factors

When comparing the performance of various encoding schemes at the different stages of the processing, several features may be analyzed, not only at a low level with the properties of the emitted codes in the baseband, but also at high level with the positioning results. Regarding the correlation functions, the simulator provides the Sidelobe-to-Mainlobe Ratio (SMR), which is the ratio between the maximum value measured outside the modulation window of the mainlobe over the peak value of the mainlobe. This is a common merit factor in CDMA-based systems [35,45] and can be used as a measurement of the difficulty to detect the correct correlation peak in order to estimate the ToA or TDoA. It can be defined as follows:

(12) $$\mathrm{SMR}=\frac{\max(C_{r,m_{k}\left[n\right]});\;\left\{\forall n\;\notin \left\{-N_{G},\;N_{G}\right\}\right\}}{\max(C_{r,m_{k}\left[n\right]});\left\{\forall n\right\}}$$

where $C_{r,m_{k}}$ represents the correlation between the received signal *r*[*n*] and the modulated code *m_k_*[*n*]; *N_G_* delimits the modulation window around the mainlobe used to avoid confusing the maximum sidelobe with the lobes corresponding to the modulation effect.

This SMR is also applied to study the goodness of codes in the baseband and after modulation, but with no channel or transmission effects; therefore, it can be divided into two merit factors: the auto-correlation bound and the cross-correlation bound. The first provides the ratio between the maximum sidelobe of the auto-correlation function and the main peak, whereas the cross-correlation bound is a measure of how two different codes are related, and is computed as the highest cross-correlation sidelobe compared with the main auto-correlation peak. In all cases, low SMR and correlation bounds are desired to clearly identify the time of arrival in the emission.

**Figure 11:**
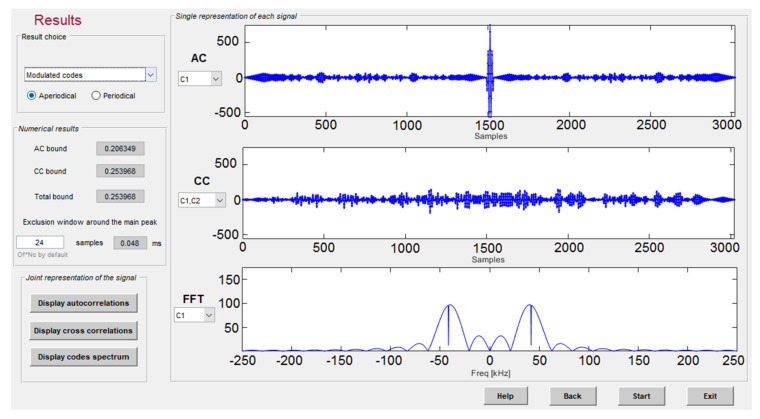
Auto-correlation and cross-correlation results for three 63-bit Kasami codes modulated in BPSK with two carrier cycles at 41.67 kHz and a sampling frequency of 500 kHz.

Figure 11 shows an example of the simulation platform, where different low-level parameters are displayed. In the example, the auto correlation and cross correlation of three 63-bit BPSK modulated Kasami codes are plotted, so it is possible to compare their performance in terms of correlation bounds and frequency spectrum with other family codes, and to study the differences in the case of periodic or aperiodic emission. In addition, the platform offers the correlation results after the modulation, with and without considering the transducers’ effects.

**Figure 12:**
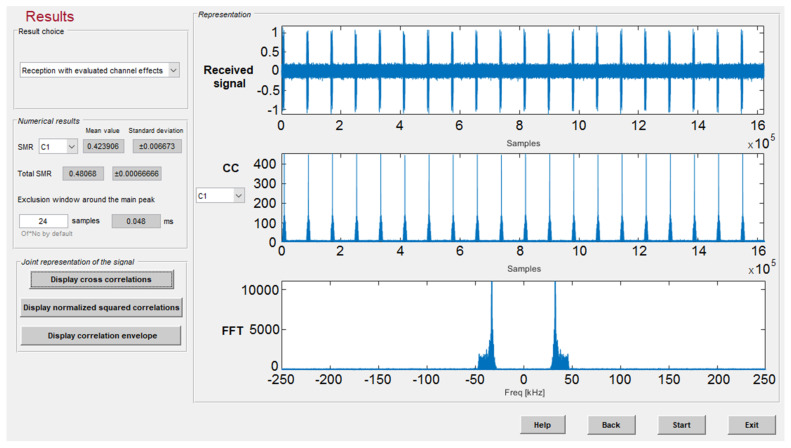
Correlation results after the emission of five simultaneous 255-bit BPSK modulated codes with SNR = 5 dB.

The correlation results involving all the effects (beacons and receiver location, transmission method, model of the transducers, and channel effects) are also displayed. The platform shows the mean value and typical deviation of the SMR for every emitter considering the number of realizations in the simulation, as can be observed in Figure 12.

**Figure 13:**
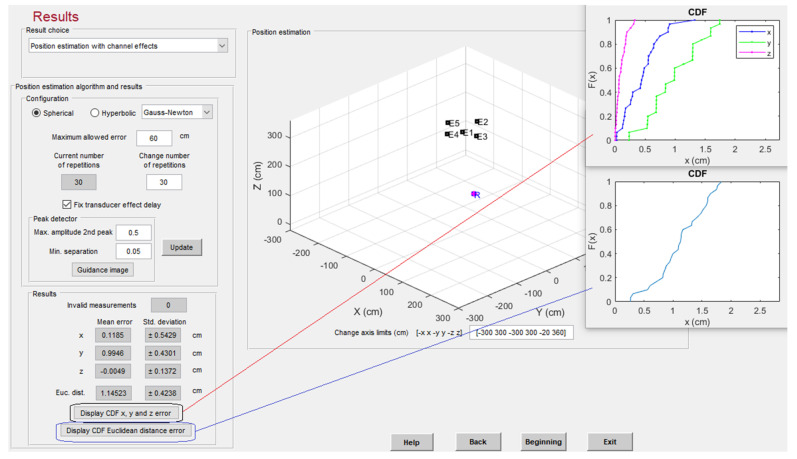
Positioning estimates obtained with 255-bit BPSK modulated codes with SNR = 5 dB, after 30 realizations.

Finally, positioning results are also presented. It is possible to choose between spherical and hyperbolic trilateration; the number of repetitions at the selected point are configurable to obtain significant statistical information, in addition to the maximum allowed error (if this error is exceeded, the measurement is considered as an outlier). The delay defined between the transducers’ emissions can be compensated for if desired. The platform provides the resulting number of outliers, the mean and standard deviation of the positioning estimates for each coordinate, and the Cumulative Distribution Function (CDF) of the Euclidian distance to the beacons, also per coordinate. The position estimates are depicted in the graph, together with the beacon distribution, as can be observed in Figure 13. It is worth mentioning that other positioning techniques, such as triangulation or hybrids, can be considered and included in CODEUS for further analysis.

## 5. Description of the CODEUS Online Demonstrator

The CODEUS online demonstrator is a remotely accessible and configurable UIPS that consists of three main blocks. First, a server allows remote access from any client to upload the configuration to be tested on the experimental demonstrator. Furthermore, this server manages a beacon unit based on a Field-Programmable Gate Array (FPGA) device and five ultrasonic transducers (E1–E5), and is thus flexible enough to implement the different required encoding and modulation schemes, and then drive the transducers. Finally, the same server also handles four receiving modules (R1–R4), which are in charge of acquiring the ultrasonic signals at some particular points in the environment where they have been placed.

**Figure 14:**
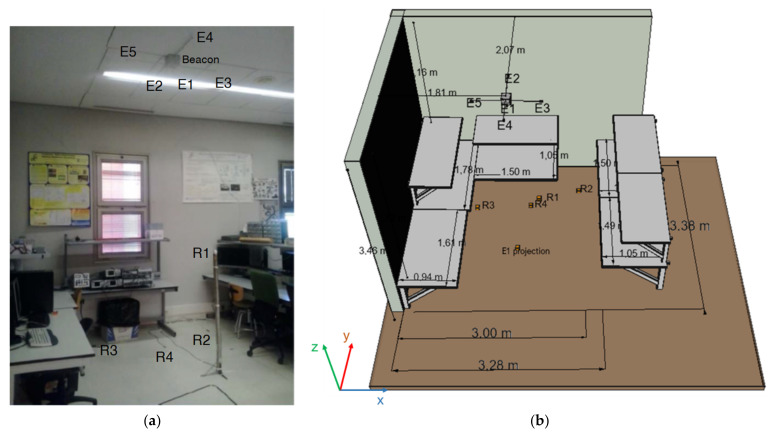
(**a**) General view of the experimental setup of the CODEUS online demonstrator, and (**b**) a scheme with the dimensions for that setup, where E1–E5 are the emitters and R1–R4 the receivers.

The CODEUS online demonstrator has been deployed in a lab environment at the School of Engineering from the University of Alcala, as can be observed in Figure 14. On the left, there is a general view of the experimental setup, denoting the beacon unit in the ceiling and the four receivers (R1–R4) in the environment. It is worth mentioning that, whereas the receiver R1 is on the top of a pole at a height of 1.34 m, the other receivers R2–R4 lie on the ground, mainly close to some furniture, which make those positions interesting from the point of view of the propagation of ultrasounds, as they are affected by multipath. Note that the origin of the coordinates of the whole setup is fixed at the projection of beacon E1 on the ground. Table 2 provides the full coordinates of the five beacons (E1–E5) and the four receivers in this experimental setup.

**Figure 15:**
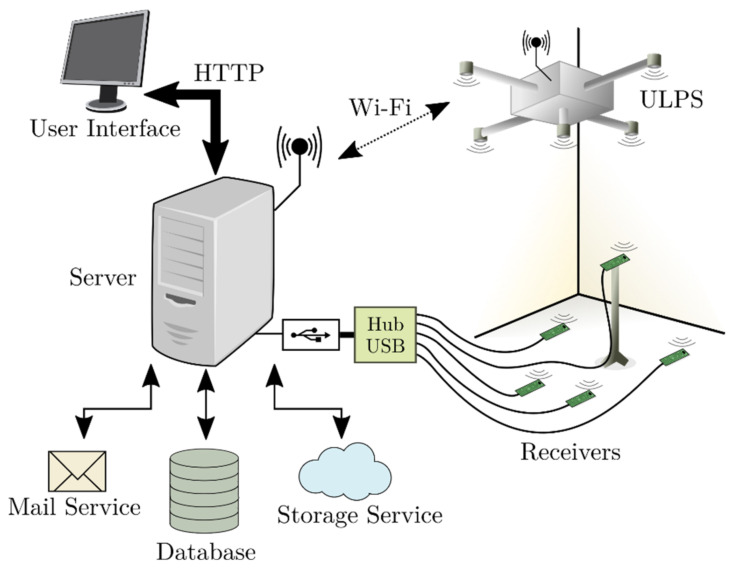
General block diagram of the server designed for the CODEUS online demonstrator.

The server provides the clients with a user-friendly interface, specified in html and php, via the HTTP protocol, so users can upload their own configuration for the five ultrasonic transmissions [64]. In this manner, it is possible to set up sequences, modulation schemes, and the repetition interval between successive transmissions. The server deals with a SQL database, by running php scripts, so the submitted configurations are stored and scheduled for their later download in the experimental setup. This download process is carried out by means of a WiFi connection between the server and the beacon unit mentioned above. This WiFi link is used by the server to open a socket and send the information to the beacon unit, again by running the corresponding php scripts. At the scheduled instant, the beacon unit starts the configured transmissions, whereas the server also manages the receiving modules to acquire the corresponding incoming signals through some USB links. These receptions will be finally uploaded to the cloud and a notification mail will be sent to the client to inform that the experimental results for their configuration are already available. Note that all the tasks developed by the server to deal with the database, the beacon unit, the receiving modules, and the cloud are implemented with php scripts. Figure 15 shows the general block diagram of the server in the CODEUS online demonstrator.

**Figure 16:**
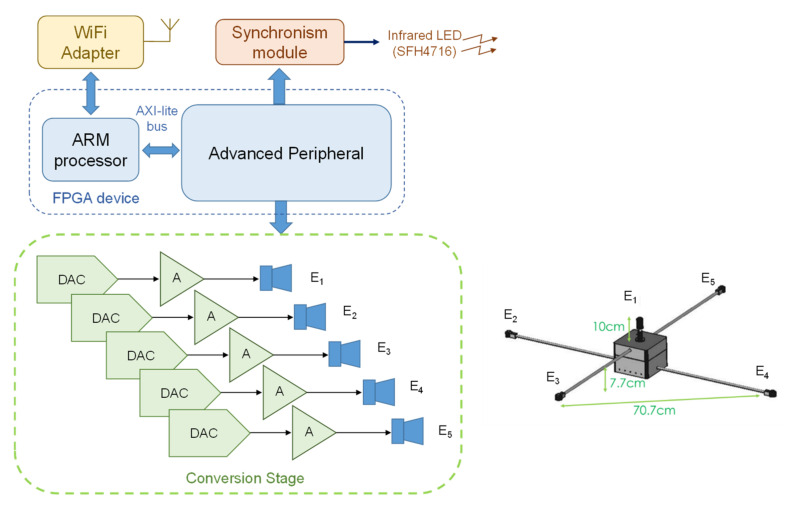
General block diagram of the beacon unit involved in the CODEUS online demonstrator [65].

With regard to the beacon unit, this module is based on a FPGA device and five Prowave 328ST160 ultrasonic emitters, as previously described in [65]. Figure 16 depicts the general block diagram of this beacon unit, and a scheme of the distribution of the five transducers *E_i_* in the beacon unit. The System-on-Chip (SoC) architecture implemented in the FPGA controls the WiFi link with the server, so it can receive configurations for the transducers’ emissions, and, afterwards, drive them suitably by means of a digital-analog converter (DAC) and an amplifier. For that purpose, an advanced specific peripheral was designed to cooperate with the ARM processor. Finally, an infrared synchronism module can be addressed by the peripheral to generate a common synchronism signal that can reach the receivers and synchronize acquisitions, in order to consider spherical positioning algorithms later.

**Figure 17:**
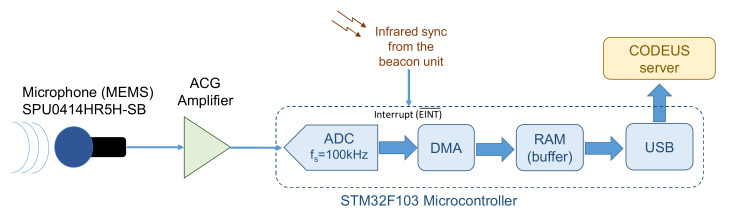
General block diagram of the receiving module included in the CODEUS online demonstrator [23].

The last part of the experimental setup of the CODEUS online demonstrator is the receiving module, whose general block diagram can be observed in Figure 17 [23]. This is based on the omnidirectional MEMS microphone SPU0414HR5H-SB (the beam divergence angle is about 180° @ −3 dB) that matches the emitters’ bandwidth around 41.67 kHz, and an analog-digital converter (ADC) together with a high-pass filter to discard audible frequencies that might saturate the ADC input and any aliasing effect. The converter is actually part of the STM32F103 processor unit, so it is in charge of sampling the incoming signals at 100 kHz. This acquisition can be synchronized with the beacons using the IR link available for that purpose. The receiver is capable of recording a data buffer with a length of 100 ms, which is uploaded to the server via the existing USB link.

## 6. Study Case of the Online Demonstrator

As a case study, this section presents some results in the particular UIPS developed in the online demonstrator, whose architectures and functionalities are described in Section 5. In order to achieve a fair comparison between simulated and experimental results, the experiments shown here with the CODEUS online demonstrator were conducted under the same configuration scheme. In this manner, 255-bit Kasami codes were involved in the transmission encoding, with a BPSK modulation with two carrier periods at a frequency *f_c_* = 41.67 kHz. One hundred acquisitions were carried out, where successive transmissions of the same beacon were separated by an interval of 100 ms and a CDMA protocol was considered, that is, all beacons transmitting simultaneously.

**Figure 18:**
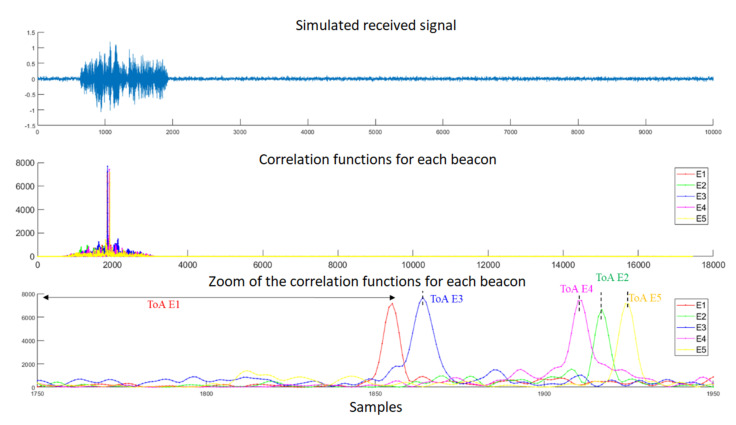
Simulated results for point R1: (**top**) The received signal with SNR = 10 dB; (**middle**) the correlation functions between this incoming signal and the codes emitted by beacons *E_i_*; (**bottom**) magnification of the area of interest, where the maximum values of those correlation functions can be observed.

**Figure 19:**
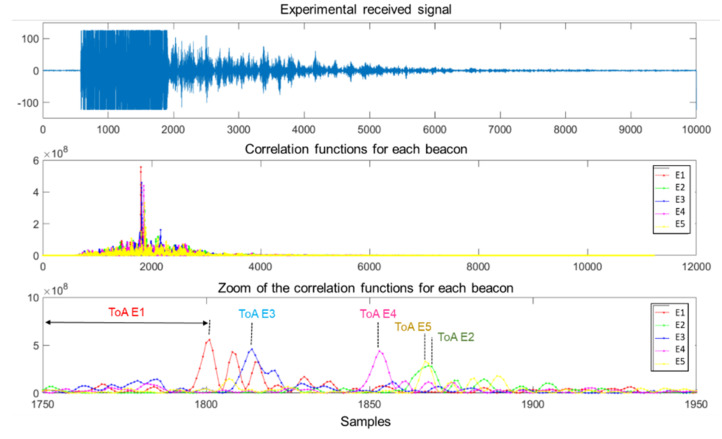
Experimental acquisition for point R1: the received signal (**top**); the correlation functions between this incoming signal and the codes emitted by beacons *Ei* (**middle**); and magnification of the area of interest, where the maximum values of those correlation functions can be observed (**bottom**).

First, Figure 18 depicts some simulated results for the test point corresponding to R1 (see Table 2), where the upper plot represents the modeled received signal and the middle plot shows the correlation functions of that incoming signal with the codes emitted by each beacon *E_i_*. For clarity’s sake, the last plot shows a zoom of those correlation functions, where the ToAs are determined by the maximum values from the mentioned correlation functions. Figure 18 can be compared with Figure 19, obtained from the experimental tests with the online demonstrator. Note that in the experimental tests, the maximum peaks indicating the ToA of the signals emitted by E2 and E5 appear to be overlapped, unlike in the simulated results, due to inaccuracies in the determination of coordinates for point R1 in the experimental scenario. In general terms, the simulated results offer lower SMR than the experimental tests carried out, so the main peaks after correlation can be better identified. This may be caused by the simplified model used to simulate the multipath, where not all of the multipath due to the furniture in the laboratory is considered, and by the fact that the experimental tests may encompass other effects that are not included in the simulation model. 

**Figure 20:**
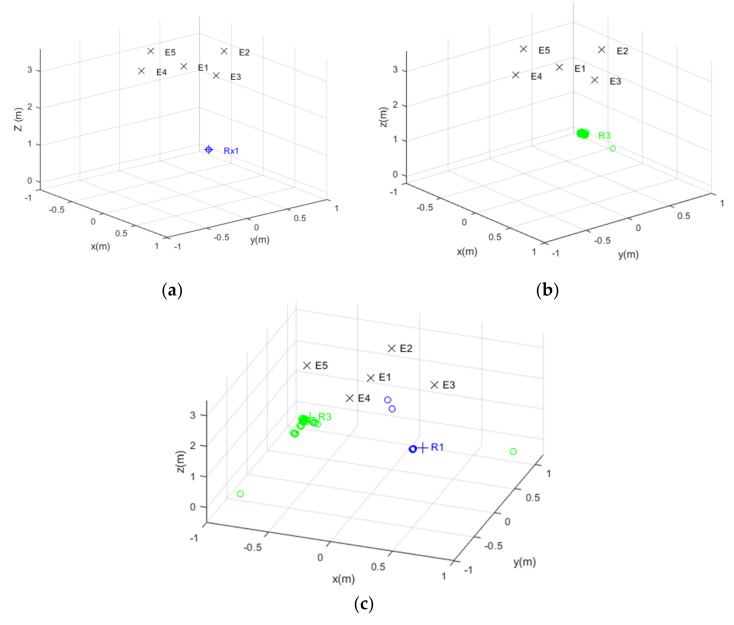
Clouds of position estimates: (**a**) simulated results for R1 with SNR = 10 dB; (**b**) simulated results for R3 with SNR = 10 dB; (**c**) experimental results for R1 and R3.

**Figure 21:**
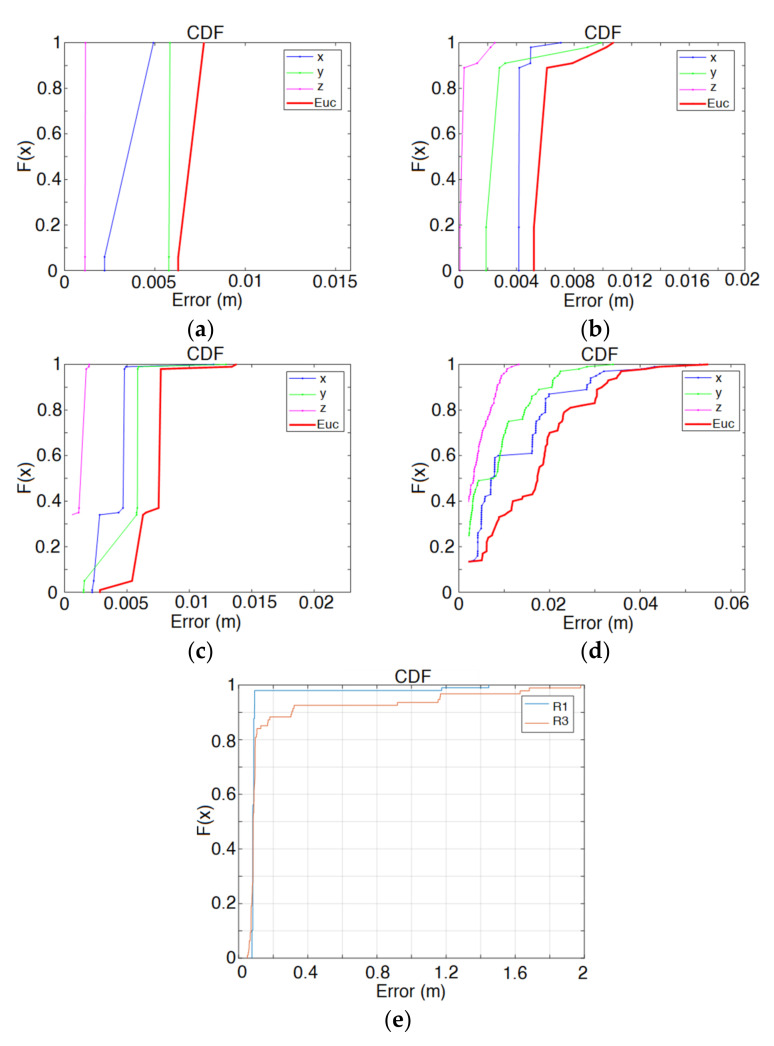
Positioning error CDF: (**a**) simulated results at R1, with SNR = 10 dB; (**b**) simulated results at R3, with SNR = 10 dB; (**c**) simulated results at R1, with SNR = 0 dB; (**d**) simulated results at R3, with SNR = 0 dB; (**e**) experimental results.

Figure 20 shows the simulated and experimental results corresponding to the clouds of estimated positions obtained for the measurement points R1 and R3, whose coordinates are shown in Table 2. Both in the simulated and experimental tests, a spherical Gauss–Newton algorithm was used to obtain the estimated positions [60]. Note that these two points were selected because R1 is a favorable case, close to the beacons and inside the common coverage area from the five beacons, whereas R3 is a challenging point, further away and with a strong influence from multipath. As can be observed, consequently, the cloud of points for R1 presents a lower dispersion (apart from a couple of outliers in the real tests in Figure 20c, likely due to a wrong determination of TDoAs on the experimental signals). Furthermore, Figure 21 shows the error CDF for both clouds of points, both in simulation and in experimental tests, where it is possible to observe that results at R1 present lower errors than at R3. In the experimental tests, 90% of measurements present a positioning error below 10 cm for R1 and below 35 cm for R3, whereas in the simulation results, errors differ by one order of magnitude. As before, the simplified model used for the simulation of the multipath in this particular environment is likely the reason for this difference, because the simulation platform does not consider the different reflectors existing in the real environment (furniture, walls, etc.) that make the situation more complex. This model should be extended in future works, in a similar manner to [35], where an image method for simulating small-room acoustics is considered. Another relevant aspect in experimental tests is that the Automatic Controlled Gain (ACG) of the receiver (see Figure 17) is required to be tuned, because the microphone voltage input is saturated in some acquisitions (see Figure 19).

## 7. Conclusions

This paper presented the CODEUS platform, a new tool that includes a highly configurable UIPS software model that is complemented with an online demonstrator. The simulator allows the study of the effect of the signal design (encoding and modulation), beacon distribution, transducers, channel effects, or the type of positioning (spherical or hyperbolic) on the correlation of the received signals and on the positioning estimates. These influences can be studied and analyzed independently for each parameter, either using the options predefined in the platform, or by incorporating customized setups. This theoretical analysis can be easily extended, by exporting the configuration to the experimental demonstrator, where the simulated results can be verified in a real environment. This CODEUS online demonstrator (freely available online: http://www3.uah.es/locate-us/ (accessed on 23 December 2021)) is a remotely accessible and configurable UIPS that allows different encoding and medium access techniques to be tested under the same conditions, easily enabling experimental tests to be carried out. Thus, the signal design can be configured with the simulation platform and real acquisitions for testing purposes can be obtained through the online demonstrator.

One of possible future extensions of the CODEUS platform is providing users with a means to integrate their own processing algorithms into the simulation platform, thus making it possible to compare the performance of different peak detectors or multipath cancellation algorithms, among others. Another aspect is the sensor fusion with other technologies, such as inertial measurements commonly available in most commercial portable devices, which can be integrated with the ultrasonic measurements by applying a Kalman filter or a particle filter. Moreover, due to the utility of acoustic signals in underwater localization of vehicles and/or sensor networks, and the challenge of deploying experimental prototypes, it is expected the tool will be extended to underwater positioning systems. Although some aspects will remain the same, such as the emission configuration, others related to the channel model require further adaptations to include the underwater propagation acoustic model, bathymetry of the seabed, temperature profile, speed of wind, water density, etc. 

## Figures and Tables

**Table 1:** Percentage of papers related to AIPS in the Indoor Positioning and Indoor Navigation Conference (IPIN).

Year	%AIPS Papers at IPIN
2016	10.32
2017	7.56
2018	9.01
2019	6.10
2021	7.69

**Table 2:** Coordinates of the five beacons (E1–E5) and the four receivers (R1–R4) in the experimental environment of the CODEUS online demonstrator.

Coordinate	E1	E2	E3	E4	E5	R1	R2	R3	R4
x (m)	0.00	0.00	0.51	0.00	−0.51	0.43	1.09	−0.80	0.19
y (m)	0.00	0.512	0.00	−0.52	0.00	−0.04	1.36	0.95	1.00
z (m)	3.29	3.43	3.37	3.43	3.37	1.34	0.00	0.00	0.00
